# Understanding Psychological Bullying in Tunisian Schools: Frequency and Determinants

**DOI:** 10.1192/j.eurpsy.2025.1102

**Published:** 2025-08-26

**Authors:** R. Ayoub, N. Maatallah, H. Ben Abid, A. Beji, A. Guedria

**Affiliations:** 1 Child and Adolescent Psychiatry Department, Fattouma Bourguiba university Hospital; 2 University of Monastir, Monastir, Tunisia

## Abstract

**Introduction:**

Psychological bullying in schools is a pervasive issue that affects the emotional and psychological well-being of countless children. This phenomenon often manifests as emotional abuse, leading to significant long-term consequences for victims.

**Objectives:**

Determine the prevalence of psychological bullying in schools in the Sousse region and to describe its associated factors

**Methods:**

This is a descriptive and analytical cross sectionnal study that took place in two middle schools and three public high schools in the Sousse region(Tunisia) among adolescents aged between 12 and 18. A pre-established data collection form enabled us to gather socio-demographic characteristics, personal history and bullying behaviours, drawing on questionnaires available in the scientific literature.

**Results:**

Our study included 420 students with an average age of 15.63±1.87 years. The sex ratio was 0.98. More than half the students (58.1%) reported being victims of bullying. The rate of harassment was higher among girls (53.7%), although this difference was not statistically significant (P=0.063). The perpetrator was a friend in 89.8% of cases, and a teacher in 35.2%. The body or physical appearance was the object of harassment in more than half of the cases. We noted that 74.1% of students with a chronic illness reported having been victims of bullying, compared to only 55.5% of students with no medical history. This difference was statistically significant (p = 0.001; OR= 2.94). This phenomenon was also noted in 81.4% of students treated for psychiatric disorders, a rate significantly higher than that of students with no psychiatric history (p<10-3; OR= 3.47).Our results showed that harassment was reported by 78.13% of adolescents living with their fathers, compared to a significantly lower proportion among those living with their mothers (51.16%) and those living with both parents (56.7%) (p = 0.008).

**Conclusions:**

Our study reveals a concerning prevalence of psychological bullying among adolescents, with over half of the participants reporting victimization. These results underscore the urgent need for targeted interventions and support systems to address bullying in schools, particularly for vulnerable populations.

**Disclosure of Interest:**

None Declared

